# Do opioids provide adequate analgesia for surgical coeliotomy in teleost fish?

**DOI:** 10.18849/ve.v10i1.702

**Published:** 2025-03-04

**Authors:** Shannen Schultz

**Keywords:** anaesthesia, analgesia, fish, opioid, pain, pain relief, surgery

## Abstract

**PICO question:**

For teleost fish undergoing surgical coeliotomy, do intramuscular exogenous opioids reduce perioperative pain, compared with no analgesia?

**Clinical bottom line:**

**Category of research:**

Treatment.

**Number and type of study designs reviewed:**

Four studies were critically reviewed. All were randomised controlled trials, 2 with a parallel group design, and 2 with a factorial crossover group design.

**Strength of evidence:**

Moderate.

**Outcomes reported:**

The studies produced contradicting results regarding the analgesic efficacy of opioids perioperatively, with some evidence of pain alleviation when assessing behavioural parameters, but not when assessing clinical pathology or quantitative cardioventilatory data.

**Conclusion:**

Perioperative use of opioids for teleost fish undergoing invasive surgical procedures may reduce behavioural changes associated with pain compared with no analgesia, but there is insufficient evidence to determine if they provide adequate pain relief based on the study methodologies.

## How to apply this evidence in practice

The application of evidence into practice should take into account multiple factors, not limited to: individual clinical expertise, patient’s circumstances and owners’ values, country, location or clinic where you work, the individual case in front of you, the availability of therapies and resources.

Knowledge Summaries are a resource to help reinforce or inform decision-making. They do not override the responsibility or judgement of the practitioner to do what is best for the animal in their care.

### Clinical Scenario

An ornamental fish keeper and avid aquarist approaches you, a first-opinion practice clinician, because one of their teleost fish has a distended abdomen, and on examination you are suspicious of an abdominal tumour. You plan to perform a coeliotomy under anaesthesia to identify the cause of the abdominal distension and want to construct an analgesic plan to go with it. You have experience with and access to opioid analgesia in your clinic, and you wish to find evidence to inform whether opioids would be an appropriate choice of perioperative analgesia in this case.

## The evidence

Four relevant laboratory-based randomised controlled trials, which are high on the hierarchy of evidence, were identified which satisfied the PICO question. All four studies investigated the effect of opioids on teleost fish undergoing surgery under anaesthesia. Baker et al. (2013) assessed changes in behaviour in fish treated with morphine, butorphanol or saline, and either with or without anaesthesia and surgery. Crivelaro et al. (2019) performed surgery under anaesthesia in all fish and compared changes in behaviour between those treated with methadone and saline. Gräns et al. (2014) assessed the impact of buprenorphine on the cardioventilatory parameters of fish either with or without surgical intervention. Harms et al. (2005) compared the impacts of butorphanol, ketoprofen, or saline on the behaviour and clinical pathology of fish undergoing surgery. There is moderate evidence that opioids provide post-surgical pain relief for teleost fish when assessing behavioural indicators of pain, but not when examining cardioventilatory data or clinical pathology.

## Summary of the evidence

**Baker et al. (2013):** Comparative analgesic efficacy of morphine sulfate and butorphanol tartrate in koi ( *Cyprinus carpio* ) undergoing unilateral gonadectomy **Aim:** To compare the effect of intramuscular morphine and butorphanol on pain-related behaviours of koi carp (Cyprinus carpio) undergoing surgical coeliotomy and unilateral gonadectomy.

Population	Adult koi ( *Cyprinus carpio*) obtained from a commercial supplier. 23 males, 19 females, and 48 of unknown sex. Mean weight 280 g. Range in length from 22.9–33.0 cm.
Sample Size	90 fish.
Intervention Details	The study period was 3.5 days. Treatment included injections of butorphanol 10 mg/kg, intramuscular (IM) (B), morphine 5.0 mg/kg, IM (M), or equivalent volume of saline solution, IM (SS). Anaesthesia was MS-222 10 mg/ml. Surgery was coeliotomy and unilateral gonadectomy. Fish were randomly allocated into three groups: Treatment only (n = 18): SS, B, M (6/group). Treatment with anaesthesia and surgery (n = 36): SS+, B+, M+ (12/group). Treatment with anaesthesia but without surgery (n = 36): SS-, B-, M- (12/group).
Study design	Laboratory-based randomised controlled trial, with factorial design.
Outcome studied	Objective variables: Respiratory rate. Food consumption. Activity level. Interactive behaviour. Hiding behaviour. Comparisons of data recorded prior to intervention with that at 0, 3, 6, 9, 12, 18, 24, 36, 48, and 72 hours postsurgery.
Main findings (relevant to PICO question)	Significant decrease in respiratory rate (P < 0.5) for all butorphanol-treated groups (B, B-, B+), as well as M+ and SS+ groups compared to baseline. B group fish had significant reductions in respiratory rate (P < 0.05) at 3, 9, 12, and 18 hours post-treatment compared to baseline, and significantly decreased (P = 0.034) respiratory rate compared to the SS group 3 hours post-treatment. B- group had significantly decreased (P < 0.05) respiratory rates between 3 and 18 hours post-treatment compared to baseline values. B+ group had significant (P < 0.05) reductions in respiratory rate at all time points from 0 to 72 hours post-treatment compared to baseline levels. M+ group had significantly decreased (P < 0.05) respiratory rates 0 to 18 hours post-treatment. SS+ group had significantly decreased (P < 0.05) respiratory rate at 12, 18, and 24 hours post-treatment. There were no changes in food consumption in treatment-only groups (SS, B, and M) compared to baseline. Surgery without analgesia (SS+) led to prolonged reduction in feeding behaviour, and surgery with butorphanol administration (B+) led to brief reduction in feeding behaviour. The SS+ group had significantly reduced feeding behaviour (P < 0.05) from 0 to 36 hours post-treatment compared to baseline. The B+ group had significantly reduced (P < 0.05) feeding behaviour at the 0 hour time point only compared to baseline. Hiding behaviour and interactive behaviour did not differ significantly between treatment groups. Water column location did not differ significantly between treatment groups or compared to baseline levels for any group. 28 of 30 (93%) of butorphanol-treated fish developed temporary buoyancy issues which resolved within 48 hr of administration. Activity levels were decreased in butorphanol injection-only group (B) compared to saline injection-only (SS). All groups receiving anaesthesia with or without surgery (SS-, SS+, B-, B+, M-, M+) had significant (P < 0.5) reductions in activity levels compared to baseline. There was a temporary hyperexcitability in 13 out of 30 (43%) morphine-treated fish Characterised by rapidly undulating in one position at the top of the water column.
Limitations	Baseline values for respiration rate and activity score were presented with mean, median, and interquartile range values, but results post-treatment were presented graphically as changes in these variables compared with baseline over time, without numerical data or descriptive statistics presented. Food consumption was also presented graphically with no numerical descriptive statistics. This makes it difficult for the reader to determine the magnitude of the clinical effect within and between treatment groups. Outcomes studied largely impacted by administration of opioids and induction of anaesthesia, and changes to these variables may represent physiological changes from these medications as opposed to reduction in pain. Single person acting as surgeon and observer, with no mention of whether this person was blinded to each fish’s intervention group when recording observations. The method of randomisation was not outlined.

Table note...

**Crivelaro et al. (2019):** Behavioural and physiological effects of methadone in the perioperative period on the Nile tilapia Oreochromis niloticus **Aim:** To assess the analgesic effect of intramuscular morphine on Nile tilapia ( *Oreochromis niloticus*) undergoing coelomic laparoscopy and surgical ventricular resection.

Population	Male adult Nile tilapia ( *Oreochromis niloticus*) *, *average weight 210.5 g, length range 18–22 cm, free from visible injury.
Sample Size	16 fish.
Intervention Details	The study period was 24 hours. Treatment included injections of methadone 30 mg/kg, intramuscular (IM), or equivalent volume of saline, IM. Anaesthesia was propofol 5 mg/ml. Surgery was laparoscopic ventricular partial resection through coelomic access. Fish were randomly allocated into two groups: Methadone group (n = 8). Control group (saline) (n = 8).
Study design	Laboratory-based randomised controlled trial, parallel design.
Outcome studied	Objective variables. Recovery time. Opercular beat rate per minute. Caudal fin beat rate per minute. Use of cover. Food consumption. Utilised recorded video data for 5-minute intervals at 1, 2, 3, 6, 12, and 24 hours post-surgery.
Main findings (relevant to PICO question)	Mean recovery time was significantly higher (P < 0.05) for methadone group (6970.0 ± 2236.0 seconds) than the control group (4569.0 ± 950.2 seconds). Ventilation rate was higher in the control group than the methadone-treated group at all time points, and mean time taken to return to spontaneous ventilation postsurgery was higher for the methadone group than the control group, but these were not statistically significant. Mean opercular beat return time was 649.9 ± 395.3 seconds for the control group, and 1284.0 ± 855.8 seconds for the methadone group but this was not statistically significant (P > 0.05). For the control group, mean ventilation rate or opercular beat rate at 1, 2, 3, 6, 12, and 24 hours postsurgery was 55.1, 44.5, 60.6, 71.1, 61.7, and 53.7 beats per minute respectively. These were higher than the methadone group, with mean ventilation rate at those times of 44.3, 49.3, 51.3, 62.0, 52.3, and 45.6 beats per minute respectively. However, this difference was not statistically significant (P > 0.05). Swimming activity was higher in control than methadone group at multiple time points, but no statistically significant differences were seen. Mean caudal fin beats per minute were higher in the control group than the methadone group at 1 hour (210.4 compared to 117.8), 2 hours (121.3 compared to 82.3), 3 hours (83.4 compared to 76.5), 6 hours (77.5 compared to 35.8), and 12 hours (51.7 compared to 38.7), but was lower in the control group compared to the methadone group at 24 hours (17.3 compared to 48.0). This difference was not statistically significant (P > 0.05). No significant differences in the mean percentage use of cover compared to baseline levels for either group (P > 0.05). No statistically significant differences in food consumption were seen between groups, more numbers of fish in control group (n = 3) returned to feed consumption within 24 hours than the methadone group (n = 1).
Limitations	Reference to basal behavioural and physiological values in discussion but these values not given. Small sample size. Assumption that caudal fin beat rate is representative of overall activity levels. The method of randomisation was not outlined.

Table note...

**Gräns et al. (2014):** Post-Surgical Analgesia in Rainbow Trout: Is Reduced Cardioventilatory Activity a Sign of Improved Animal Welfare or the Adverse Effects of an Opioid Drug? **Aim: **To assess the effect of intramuscular buprenorphine on the ventilation and heart rate of rainbow trout ( *Oncorhyncus mykiss*) undergoing surgical coeliotomy.

Population	Rainbow trout ( *Oncorhynchus mykiss*) obtained from a local hatchery. Size range 500–1048 g, mean 743 g.
Sample Size	36 fish.
Intervention Details	The study period was 9 weeks in total, with each fish monitored for 7 days after their experimental procedure. Treatment (Tx) was buprenorphine 0.05 mg/kg, intramuscular (IM). Anaesthesia was MS-222 150 mg/l. Surgery (Sx) was Fish were randomly allocated into four groups, with 9/group: Anaesthesia with no surgery or treatment (Sx(-)Tx(-)). Anaesthesia with treatment but no surgery (Sx(-)Tx(+)). Anaesthesia with surgery but no treatment (Sx(+)Tx(-)). Anaesthesia with surgery and treatment (Sx(+)Tx(+)).
Study design	Laboratory-based randomised controlled trial, factorial design.
Outcome studied	Objective assessment. Cardioventilatory data was monitored for 7 days postoperatively. Electrodes in the water detected bioelectric potentials generated from cardioventilatory muscle activity, which was amplified and processed to record ventilation rate, heart rate, and heart rate variability. Data was recorded for 4 hours each day, from 12am–4am, at times of least spontaneous muscle activity.
Main findings (relevant to PICO question)	Buprenorphine significantly decreased (P < 0.05) heart rate and ventilation rate compared with the control group across all 7 days. Surgical intervention did not significantly impact ventilation or heart rate in the buprenorphine treated groups.
Limitations	Data for heart rate, heart rate variability, and ventilation rate were presented graphically with no numerical data to allow direct comparisons, and no descriptive statistics, which makes it challenging to determine the magnitude of the impact of treatment. It is challenging for the reader to determine which results are of statistical significance due to their graphical presentation, with icons used to denote which days and groups showed significant differences. It was also noted in the methods that differences with P < 0.05 were deemed statistically significant, but specific values were not given for each comparison. Recording period was for 4 hours daily from 12am–4am and does not describe physiological changes in the immediate postoperative period, or at differing time points. Over a 9-week period, 4 fish per week were involved allowing for possible experimental variation over time. The two groups that did not receive treatment (Tx(-) groups) did not receive an equivalent volume placebo such as saline. No mention of blinding in study methods. The method of randomisation was not outlined.

Table note...

**Harms et al. (2005):** Behavioral and clinical pathology changes in koi carp (Cyprinus carpio) subjected to anesthesia and surgery with and without intra-operative analgesics **Aim: **To compare the analgesic effect of intramuscular butorphanol, an opioid, and ketoprofen, a non-steroidal anti-inflammatory analgesic, on the behaviour and clinical pathology of koi carp ( *Cyprinus carpio*) undergoing surgical coeliotomy.

Population	Koi carp ( *Cyprinus carpio*), 20 male, 8 female, 2 sex unknown, obtained from commercial producer. Size range 58–265 g, mean 126 g.
Sample Size	30 fish.
Intervention Details	The study period was 16 days, with blood samples acquired 14 days before surgery, and observations made for 48 hours postsurgery. Treatment included intramuscular (IM) injection of butorphanol 0.4 mg/kg, ketoprofen 2 mg/kg, or equivalent volume of saline. Anaesthesia was MS-222 100–200 mg/l. Surgery was exploratory coeliotomy. Fish were randomly allocated into three groups (10/group): Butorphanol Ketoprofen Control group (saline).
Study design	Laboratory-based randomised controlled trial, parallel design.
Outcome studied	Objective assessment. Vertical position in water column (5 cm increments). Activity level (caudal fin beat rate per minute). Respiratory rate. Response to food. Clinical pathology. Haematocrit, total solids, plasma cortisol, and complete plasma biochemistry panels were compared between presurgical and postsurgical samples.
Main findings (relevant to PICO question)	The butorphanol-treated group was the only one to not have significant decreases in caudal fin beat rate and vertical column position. Median vertical position in the water column for all fish was 15 cm above the bottom of the tank presurgery, with a median low of 10 cm at 0.5 and 18 hours postsurgery, with significant decreases (P < 0.05) at 0.5, 1, 18, and 48 hours postsurgery for all fish. No significant change in vertical position for butorphanol-treated group at any time point, with significant decreases in ketoprofen-treated group at 1, 2, and 18 hours postsurgery, and saline-treated (control) group at 0.5 hours postsurgery only (P < 0.05). There are no reported changes in vertical position for the control group after 0.5 hours postsurgery. There were significant (P < 0.05) decreases in caudal fin beat rate at 0.5, 2, 3, 6, and 18 hours postsurgery for all fish. Median presurgery caudal fin beat rate was 52 beats per minute, with a median postsurgery rate of 38 beats per minute. Respiratory rate was decreased when comparing postsurgical values to baseline values, but there was no significant difference between groups. Significant decrease (P < 0.05) in respiratory rate for all fish at 6 and 18 hours postsurgery compared to presurgery values. Median respiratory rate for all fish was 28 breaths per minute presurgery, compared with a median of 14 breaths per minute postsurgery. Fewer fish consumed the feed pellet in the hour after surgery compared with other time points, but there was no significant difference between groups. At 0.5 and 1 hour postsurgery, 21 and 24 fish respectively consumed the feed pellet, compared with 28–30 fish at all other time points. Comparison between groups was unreliable due to the overall low rate of feed refusal at all time points. The only clinical pathological difference between treatment groups was a lower increase in creatinine kinase (CK) in the ketoprofen-treated group compared with the butorphanol-treated group or control. CK increased by 90 u/litre in the ketoprofen-treated group after surgery, significantly less than the butorphanol-treated (2467 u/litre increase) and saline-treated (2267 u/litre) groups (P < 0.05). For all groups, compared to presurgical values there were significant (P < 0.05) postsurgical decreases in median haematocrit (25% compared with 36%), total solids (1.8 g/dl compared with 2.2 g/dl), phosphorus (5.3 mg/dl compared with 6.4 mg/dl), total protein (1.9 g/dl compared with 2.0 g/dl), potassium (3.0 mmol/l compared with 3.4 mmol/l), and chloride (111 mmol/l compared with 115 mmol/l). For all groups, compared to presurgical values there were significant (P < 0.05) postsurgical increases in median plasma glucose (82 mg/dl compared with 36 mg/dl), aspartate transaminase (AST) (600 u/l compared with 103 u/l), CK (5658 u/l compared with 3387 u/l), and bicarbonate (8 mmol/l compared with 6 mmol/l).
Limitations	Assumption that decreased caudal fin beat rate is representative of decreased overall activity levels. Haematology and biochemistry results obtained by comparing presurgical and postsurgical values, not considering normal species reference ranges. The method of randomisation was not outlined.

Table note...

## Appraisal, application and reflection

There has been a recent surge in demand for successful methods of surgical analgesia for teleost fish. Though there has been debate regarding whether fish neuroanatomy can allow for interpretation of nociception, it has been shown that fish possess nociceptors. Additionally, when presented with noxious stimuli, they display physiological responses such as changes to opercular rate and heart rate and develop avoidance behaviours (Sneddon, 2012). Teleost fish have been involved with invasive surgical procedures as part of biomedical research, aquaculture, and ornamental fish medicine for decades, but postsurgical analgesia is seldom a feature of fish anaesthetic protocols. To maximise animal welfare and optimise postoperative recovery, adequate analgesia should be used which limits behavioural and physiological changes when compared with presurgical values. The opioid class of analgesics are effective at attenuating these changes in response to nonsurgical noxious stimuli in fish and could be beneficial in the perioperative period for painful surgical procedures (Stoskopf & Posner, 2008). The goal of this Knowledge Summary was to investigate if current evidence supports the use of opioids as part of the surgical anaesthetic plan for these fish species.

Current evidence suggests that the most effective option for analgesia in fish are those in the opioid class, particularly mu-opioid agonists (Sladky, 2023). One of the challenges associated with determining effective analgesic plans in fish is the vast interspecies diversity regarding anatomy, physiology, and pharmacology. Species differences can influence the pharmacokinetics and pharmacodynamics of analgesic drugs, such as with the slower metabolism of morphine in winter flounder compared with rainbow trout (Chatigny et al., 2018), and the significant difference in maximum plasma morphine concentration between goldfish and salmon (Nordgreen et al., 2009). It is important to therefore make comparisons between similar species where possible, and that appropriate species selection is carried out when performing research, so that results may be reliably applied. Interspecies differences may also influence the route of administration of analgesic drugs. Administering analgesia via immersion bath, which exposes the gills to a prescribed dose of a soluble drug, can be an effective administration technique, particularly for small fishes in which injection may prove difficult. Intracoelomic or intraperitoneal injection allows for large volumes to be administered, particularly in larger fishes but carries risk of injecting organs and may have reduced absorption compared with intramuscular injection. Intravenous injection is not routinely used in fish due to the intricate nature of their blood vessels (Sladky, 2023). In the appraised studies, there is also wide variety of concentrations of anaesthetic agent used, which may impact on the reliability of results and comparisons between them. It is therefore crucial to compare studies that involve a similar method of drug administration in a similar species to make reliable conclusions.

While the goal of this Knowledge Summary was to assess the literature relating to opioid use for surgical coeliotomy from a clinical perspective, much of the literature related to less-invasive noxious stimuli in laboratory settings and the results of these studies should be acknowledged. Surgical fin clipping is a common procedure in research animals, particularly zebrafish, and has been shown to be a painful procedure leading to increased respiratory rate, lower tank position, and reduced activity levels (Schroeder & Sneddon, 2017), as well as reductions in swimming speed and tank exploration (Deakin et al., 2019). Immersion therapy with methadone reduced these behavioural changes (Deakin et al., 2019), but immersion in butorphanol did not provide significant differences in these parameters compared with a control group (Schroeder & Sneddon, 2017). The studies assessing analgesia for fin clipping may be extrapolated to other surgical procedures such as lumpectomies, but more research in this area would be beneficial.

Due to the common practice of injecting laboratory animals with drugs in research, a large amount of literature focuses on pain associated with injection of noxious chemicals into various locations in fish. Acetic acid is commonly used as a pain-inducing chemical and can cause rubbing of the area injected in rainbow trout (Sneddon, 2004) and catfish (Rodrigues et al., 2019), as well as increased circling and abdominal writhing behaviours (Costa et al., 2023), and changes to locomotory activity in zebrafish (Taylor et al., 2017; Lopez-Luna et al., 2017). Acetic acid injection also reduced interactive group and social behaviours in zebrafish (Rosa et al. 2022). Similar nociceptive effects can be seen with administration of formalin in zebrafish (Magalhâes et al., 2017) and catfish (Rodrigues et al., 2019). The administration of morphine reduced these nociceptive changes in all cases. While very relevant in a laboratory setting, the injection of noxious chemicals is less relevant to a clinical or therapeutic setting.

There are other noxious stimuli that are assessed in pain studies in fish that may not be applicable to the clinical setting, namely electricity and corneal noxious stimulation. Application of an electric shock decreased locomotor activity in zebrafish larvae, which was minimised by treatment with buprenorphine prior to electric stimulus (Steenbergen, 2018). An electric shock produced an agitated swimming response in goldfish, which was inhibited with administration of morphine (Ehrensing et al., 1982). Additionally, an electric shock to the face region caused changes to fin and tail movement in rainbow trout, and these changes were minimised by treating with morphine in a dose-dependent manner (Jones et al., 2012). Application of hypertonic saline to the cornea of zebrafish resulted in a significant change in locomotory activity associated with nociception. This effect was inhibited with administration of morphine, which in turn was inhibited by the administration of the opioid-antagonist naloxone (Magalhâes et al., 2018).

A major consideration when interpreting the results of these studies is the effect of the endogenous analgesic system on antinociception. When piauçu were injected with 3% formaldehyde as a noxious stimulus, there was an increase in locomotor activity. When the fish were exposed to stress by either restraining them (Wolkers et al., 2013), or exposing them to a conspecific alarm substance (Alves et al., 2013), which is a fear-evoking epidermal-released chemical used to signal predatory risk to other animals (Li et al., 2023), this change in locomotor activity was reduced, suggesting a possible antinociception that is restraint or stress-induced. When the opioid antagonist naloxone was given prior to restraint, this inhibition was blocked, indicating involvement of opioid receptors in stress-induced antinociception (Wolkers et al. 2013; Alves et al. 2013). Additionally, zebrafish undergoing fin-clipping surgery exhibited fewer pain-associated behavioural changes when first exposed to acute or chronic stress. When naloxone was administered prior to the procedure and the stress, zebrafish exhibited similar behavioural changes to the stress-free zebrafish (Thomson et al., 2020). Furthermore, when zebrafish were injected with acetic acid, a noxious stimulus, in conjunction with naloxone, the pain response exhibited by increased abdominal curvature was prolonged, indicating the role of endogenous opioids in the recovery from pain (Costa et al., 2019). It is important to consider the possible effects of stress-induced analgesia when interpreting the results of these studies, particularly when repeated conscious handling and restraint is part of the study design. It is advisable that assessment of cortisol levels is performed to ensure stress is not a confounding factor (Jones et al., 2012).

Allocation of participants to treatment groups is described as random in all four studies, but the method of randomisation was not outlined. It is therefore not clear that the authors truly minimised confounding factors with successful randomisation. Three out of four studies specified that they were blind studies, reducing possible biases. In Baker et al. (2013), a single person acting as surgeon and observer was blinded to which injection was administered. In Crivelaro et al. (2019), the observer of recovery period videos was blinded to the treatment group, but it was not outlined whether surgeons were blinded to treatment prior to procedure. In Harms et al. (2005), surgeons were blinded to the treatment group, as treatments were made up to equivalent volumes for body weight, and observers were blinded to both treatment and group. But in Gräns et al. (2014), there was no mention of blinding in the article, and it is not clear whether the observer was aware of the intervention and treatment group of participants.

Ideally the outcomes reported for each study would be based on species-specific guidelines outlining the most reliable indicators of pain, but this type of information is available for very few species. Zebrafish are the subject of many research studies, and as such more detailed data is available describing the most appropriate outcomes when assessing pain in these animals. Measuring activity levels, total distance travelled, and utilisation of space can be reliable indicators of pain in zebrafish (Sneddon et al. 2023). There are also automated methods of measuring these variables, such as the Fish Behaviour Index (FBI), which uses cameras with automated tracking software to automatically measure distance travelled and activity levels at different time points during the study (Deakin et al., 2019). There are concerns about the applicability of these behavioural indicators of pain in zebrafish to other less commonly used species, such as the koi carp, rainbow trout, and Nile tilapia utilised in the appraised studies. There are currently no standardised measures of pain in these species, and further research should be done to create similar guidelines for other common species, so that there can be uniformity in outcome selection between studies.

All four studies involved assessment of behavioural or physiological parameters in the postsurgical period. Baker et al. (2013) compared data recorded prior to intervention with that at multiple intervals postsurgery. Outcomes assessed were respiratory rate, food consumption, activity level, interactive behaviour, and hiding behaviour. Respiratory rate, food consumption, and activity level were found to be the most useful indicators of surgical pain in koi carp, when compared to baseline levels.

Crivelaro et al. (2019) utilised recorded video data for 5-minute intervals at 1, 2, 3, 6, 12, and 24-hours postsurgery. Opercular beat rate, caudal fin beat rate, use of cover, and food consumption were measured. The authors did not find that any of these outcomes reliably indicated surgical pain or its alleviation with analgesia, but the outcomes were only compared between the exposure and control groups and were not compared with presurgical or baseline values. Therefore, behavioural and physiological variation between treatment groups could be confounding results and downplaying their significance.

Harms et al. (2005) measured vertical position in water column, activity level, respiratory rate, and response to food as key outcomes. Additionally, for assessment of clinical pathology, haematocrit, total solids, plasma cortisol, and complete plasma biochemistry panels were compared between presurgical and postsurgical samples. The authors report that vertical position in the water column, activity level, and food response were indicators of surgical pain. Clinical pathology changes were consistent with surgical haemorrhage, and minimal differences were seen between profiles of participants in either treatment group. But haematology and biochemistry profiles were compared only between presurgical and postsurgical samples, and were not compared with species-specific reference ranges, which may place these results within normal limits.

Gräns et al. (2014) was the only study to not use behavioural or observational outcomes in their trial, instead relying on quantifiable data. Cardioventilatory data was monitored for 7 days postoperatively. Electrodes in the water detected bioelectric potentials generated from cardioventilatory muscle activity, which was amplified and processed to record ventilation rate, heart rate, and heart rate variability. But data was recorded for only 4 hours each day, from 12am–4am, at times of least spontaneous muscle activity. Therefore, the immediate postoperative period was not involved, nor were varying time points throughout the day. Additionally, by removing direct observation and behavioural data from the trial, a full assessment may have missed the analgesic effects of the treatment.

In all of the appraised studies (Baker et al., 2013; Crivelaro et al., 2019; Harms et al., 2005; and Gräns et al., 2014), many of the outcomes such as activity level and respiration rate may be affected by the sedative effect of opioids and their tendency to cause cardiorespiratory depression. A profound bradycardia and reduction in cardiac output, as well as a transient decrease in respiratory rate were observed after treatment of morphine in winter flounder (Newby et al., 2007). Similarly, administration of fentanyl to zebrafish larvae resulted in a marked reduction in respiratory rate, which was inhibited by the opioid-antagonist naloxone (Zaig et al., 2021). Gräns et al. (2014) and Baker et al. (2013) attempted to account for this by assessing variables in fish receiving opioids with and without surgery. Without this distinction, it is difficult to determine whether the outcomes were affected by a reduction in pain after administration of opioids, or by adverse effects of the opioids themselves.

Baker et al. (2013) found that both butorphanol and morphine reduced behavioural changes in koi carp undergoing coeliotomy and unilateral gonadectomy. Similarly, Harms et al. (2005) also reported that treatment with butorphanol led to no significant decreases from presurgical behaviours in koi carp undergoing exploratory coeliotomy. These authors concluded that opioids may have some analgesic effect perioperatively for these animals. However, Crivelaro et al. (2019) suggest that methadone may not have an analgesic effect, but merely a sedative effect on Nile tilapia undergoing laparoscopic ventricular partial resection. Finally, Gräns et al. (2014) proposed that buprenorphine causes nondiscriminatory cardiovascular and respiratory depression in rainbow trout, whether undergoing coeliotomy or not.

Within the appraised studies, there is a great deal of variation in species used, opioid intervention administered, and outcomes assessed. Further research needs to be undertaken with a consistent approach to measuring opioid efficacy and focusing on appropriate dosages required to provide surgical analgesia. This should be done by focusing on determining the physiologic impact of opioids on teleost fish so that their analgesic effects can be more accurately assessed, as well as development of more uniform measures of pain in these species. Although there were differences in the studies’ findings regarding the true analgesic efficacy of opioids for teleosts undergoing surgery, and their possible adverse effects, the results of the trials indicate that there is room for opioid analgesia in the anaesthetic protocols for these animals.

## Methodology

**Search Strategy:** Caption placeholder

Databases searched and dates covered	CAB Abstracts on the OVID interface 1973 to January 2024 Scopus (via Elsevier) 1970 to January 2024 PubMed on the NCBI interface 1967 to January 2024
Search terms	CAB Abstracts: (fish or teleost).mp. (opiate* or opioid* or morphine or methadone or butorphanol or buprenorphine).mp. (surgery or surgical).mp. analgesi*.mp. 1 and 2 and 3 and 4 Scopus: ( title-abs-key ( fish OR teleost ) AND TITLE-ABS-KEY ( opiate OR opioid OR morphine OR methadone OR butorphanol OR buprenorphine ) AND TITLE-ABS-KEY ( surgery OR surgical ) AND TITLE-ABS-KEY ( analgesia OR analgesic ) ) PubMed: (fish OR teleost) AND (opiate OR opioid OR morphine OR methadone OR butorphanol OR buprenorphine) AND (surgery OR surgical) AND (analgesia OR analgesic)
Dates searches performed:	15 Jan 2024

Table note placeholder

**Exclusion/Inclusion Criteria:** Caption placeholder

Exclusion	Investigating non-fish species. Not primary studies (case reports, literature reviews). Surgical coeliotomy not included in study design.
Inclusion	All included studies involved inducing anaesthesia and performing an invasive surgical coeliotomy, with an injectable exogenous opioid analgesia administered during the perioperative period. All studies included assessment of behavioural and/or physiological parameters in the postsurgical period.

Table note placeholder

**Search Outcome:** Caption placeholder

Database	Number of results	Excluded –irrelevant species	Excluded – study design(literature review or case report)	Excluded – lack ofcolieotomy surgery	Total relevant papers
CAB Abstracts	29	18	3	4	4
Scopus	21	10	5	2	4
PubMed	9	1	3	2	3
Total relevant papers when duplicates removed					4

Table note placeholder

## ORCID

Shannen Schultz: https://orcid.org/0009-0009-2835-3936

## Conflict of interest

The authors declare no conflicts of interest.

## Disclaimer

Knowledge Summaries are a peer-reviewed article type which aims to answer a clinical question based on the best available current evidence. It does not override the responsibility of the practitioner. Informed decisions should be made by considering such factors as individual clinical expertise and judgement along with patient’s circumstances and owners’ values. Knowledge Summaries are a resource to help inform and any opinions expressed within the Knowledge Summaries are the author’s own and do not necessarily reflect the view of the RCVS Knowledge. Authors are responsible for the accuracy of the content. While the Editor and Publisher believe that all content herein are in accord with current recommendations and practice at the time of publication, they accept no legal responsibility for any errors or omissions, and make no warranty, express or implied, with respect to material contained within. For further information please refer to our Terms of Use.
